# Supplementary material for: Travelers’ Attitudes, Behaviors, and Practices on the Prevention of Infectious Diseases: A Study for Non-European Destinations
Source: Int J Environ Res Public Health. 2021 Mar 18;18(6):3110. doi: 10.3390/ijerph18063110 (PMC8002973; doi:10.3390/ijerph18063110)
Supplement: Supplementary file 1 [file ijerph-18-03110-s001.pdf]

International Journal of
Environmental Research
and Public Health

MDPI

File S1: Questionnaire

- Age group:

[ ] 18–24; [ ] 25–34; [ ] 35–44; [ ] 45–54; [ ] 55–64; [ ] >64;

- Gender: [ ] M [ ] F

- Citizenship: [ ] Italian; [ ] Foreign; [ ] Others

- Actually, do you live in Italy? [ ] Yes; [ ] No

- Occupation: [ ] Student; [ ] Worker; [ ] Houseworker; [ ] Unemployed; [ ] Retired

- Education: [ ] Elementary diploma; [ ] Secondary school education; [ ] High school diploma; [ ] University degree; [ ] Postgraduate qualification

- Have you ever travelled outside the European Union? [ ] Yes; [ ] No

- Do you intend to make an international trip outside the European Union in the future? [ ] Yes; [ ] No

- Generally, for what reasons do you travel? [ ] For work; [ ] For Study; [ ] Voluntary work; [ ] Leisure/tourism; [ ] To visit relatives and friends

- How long before the departure, do you plan your travel? [ ] One month or more; [ ] Between one month and 2 weeks before; [ ] Less than two weeks before; [ ] Only one week before

- Do you seek information about travel health risks before the departure? [ ] Yes; [ ] No

- If so, what are your information sources? [ ] Health professional; [ ] Family or friends; [ ] Internet; [ ] Embassies or consulates; [ ] Vaccination clinics or travelers medical clinic; [ ] Books; apps; [ ] Travel agency; [ ] Pharmacist; [ ] Social networks

- Did you refuse or would refuse recommended vaccinations before a travel outside the European Union? [ ] Yes; [ ] No; [ ] Do not know

- If so, why did/would you refuse them? [ ] No enough time; [ ] Not useful; [ ] Fear of collateral effects; [ ] cost; [ ] Difficulties in gaining access to vaccination clinic; [ ] Other

- When you travel, do you pay attention to the following products?

[ ] Raw fruits and vegetables; [ ] Tap water; [ ] Ice cubes; [ ] Bottled water; [ ] Raw seafood
